# Health of quilombola children as a challenge for the Sustainable Development Goals: a scoping review

**DOI:** 10.1590/0034-7167-2024-0106

**Published:** 2025-01-10

**Authors:** Emanuella Pereira de Lacerda, Sara Fiterman Lima, Bruno Luciano Carneiro Alves de Oliveira

**Affiliations:** I Universidade Federal do Maranhão. São Luís, Maranhão, Brazil

**Keywords:** Child, Child Health, Black People, Quilombo, Quilombola Communities, Niño, Salud Infantil, Población Negra, Quilombo, Quilombola

## Abstract

**Objective:**

to map the literature on quilombola children’s health and its relationship with the Sustainable Development Goals.

**Method:**

a scoping review, which followed the JBI protocol and the Preferred Reporting Items for Systematic reviews and Meta-Analyses extension for Scoping Reviews. Searches were conducted in the LILACS, BDENF, Web of Science, Scopus, MEDLINE databases and Google Scholar platform. The research protocol was registered in the Open Science Framework.

**Results:**

eighteen articles out of 2,055 studies were selected as relevant for this study. The articles were grouped into four axes: Access to healthcare services; Nutritional aspects of quilombola children; Health problems of quilombola children; and Care for quilombola children. The relationship between these articles and SDGs 1, 3, 4, 6 and 10 was observed.

**Final considerations:**

the study provided an extremely important mapping of the theme of quilombola children’s health and themes related to the Sustainable Development Goals.

## RESUMEN

**Objetivo:**

mapear la literatura sobre la salud de los niños quilombolas y su relación con los Objetivos de Desarrollo Sostenible.

**Método:**

revisión de alcance, que siguió el protocolo del JBI y los elementos de informe preferidos para revisiones sistemáticas y la extensión de metaanálisis para revisiones de alcance. Las búsquedas se realizaron en las bases LILACS, BDENF, Web of Science, Scopus, MEDLINE y plataforma Google Scholar. El protocolo de investigación fue registrado en el Open Science Framework.

**Resultados:**

se seleccionaron 18 artículos de 2.055 estudios como relevantes para este estudio. Los artículos fueron agrupados en cuatro ejes: Acceso a los servicios de salud; Aspectos nutricionales de los niños quilombolas; Problemas de salud de los niños quilombolas; y Atención a los niños quilombolas. Se observó la relación entre estos y los ODS 1, 3, 4, 6 y 10.

**Consideraciones finales:**

el estudio proporcionó un mapeo extremadamente importante de la salud de los niños quilombolas y de temas relacionados con los Objetivos de Desarrollo Sostenible.

## INTRODUCTION

The Sustainable Development Goals (SDGs), first launched in 2015 during the United Nations General Assembly, aim to end poverty, protect the planet and ensure that all people enjoy peace and prosperity. The SDGs consist of four main dimensions (social, environmental, economic and institutional) and 17 universal goals, which are relevant to *quilombola* (common name for slaves who took refuge in *quilombos*, or descendants of black slaves whose ancestors, during the period of slavery, fled from sugar cane mills, farms and small properties where they performed various manual labor tasks to form small villages called *quilombos*) communities, which face social, economic and environmental inequalities^(1,2)^.

Regarding *quilombola* children, a direct relationship with the following SDGs can be highlighted: SDG 1, to eradicate poverty in all its forms, everywhere, as many live in situations of poverty and with limited access to basic resources; and SDG 3, which seeks to ensure a healthy life and promote well-being for all at all ages, considering possible disparities such as lack of access to basic care and poor sanitation conditions.

Considering possible disparities such as lack of access to basic care and poor sanitation conditions, the following SDGs stand out: SDG 4, which focuses on quality education for all, considering potential challenges due to structural and cultural inequalities; SDG 6, which aims to ensure the availability and sustainable management of water and sanitation for all; SDG 10, which aims to reduce economic, social and political inequalities, as *quilombola* communities, including children, often face high levels of social and economic inequality^(3,4)^.

Recently, the *Quilombos* and Education Project, developed by the *Coordenação Nacional de Articulação das Comunidades Negras Rurais Quilombolas* (CONAQ, National Coordination for the Articulation of Black Rural *Quilombola* Communities)^(5)^, has been addressing the social and health needs of *quilombola* children in Brazil, given that the country has approximately 151 thousand students enrolled in 1,253 schools located in *quilombola* communities, which reveals a considerable population^(6)^. Despite this, *quilombola* children’s living and health conditions still receive little attention given the magnitude of this issue, and available studies reveal significant public health problems, such as quality of life, basic sanitation problems and difficulties in accessing social and health services, especially among the extremes of age, such as older adults and children^(7)^. Although these results are clearly related to the SDGs, many of these studies do not establish this relationship.

It is important to highlight the interface with nursing, since there is a close connection between this area and the SDGs, revealed from daily work, in actions committed to promoting the health and well-being of those under their care, in all age groups and in different socio-environmental contexts, playing a vital role and transforming global goals into tangible actions and promoting a healthier, more equitable and sustainable world ^(8)^.

In this context, it is understood that this scoping review becomes relevant, since it allows, in addition to the survey of scientific evidence, to highlight *quilombola* children’s health and its correlation with the SDGs, especially SDG 3, which deals with health and well-being. In this way, it allows a differentiated look at the topic and a reflection on the inclusion of this population in agendas that aim at changes for specific communities, such as *quilombola* children.

## OBJECTIVE

To map the literature on *quilombola* children’s health and its relationship with the SDGs.

## METHODS

### Ethical aspects

As this is a scoping review study, this study does not require assessment by a Research Ethics Committee.

### Study design

This is a scoping review, which aims to identify scientific evidence in the literature on *quilombola* children’s health and especially their relationship with the SDGs, especially SDG3: Good health and well-being.

### Methodological procedure

For the review, the methodological approach followed the JBI protocol^(9)^, which is a guide for scoping review. The methodological instrumentation occurred through an organization that was prepared in a spreadsheet editor, namely Microsoft Office Excel, with the execution of four stages, which were carried out in June and July 2023, namely: 1) research question identification; 2) identification of relevant studies; 3) study selection; 4) study analysis; and 5) data grouping, synthesis and presentation.

The research protocol with definition of criteria, transparency and replication was registered on the Open Science Framework platform, under identifier 10.17605/OSF.IO/37SU5^(10)^. The study question was formulated according to the acronym Participants, Concept, Context (PCC). For this study, P refers to *quilombola* children, C to health, and C to world. The study questions were: what topics related to *quilombola* children’s health are available in the literature? What aspects of *quilombola* children’s health are in light of the SDGs? The next stage was to search the databases through institutional access. The third stage was to select a controlled vocabulary from the Health Sciences Descriptors (DeCS), the Medical Subject Headings Section (MeSH) and EMTREE, as shown in Chart 1.

**Chart 1:** Location of terms for the study

	Terms in Portuguese	Terms in Spanish	Terms in English
DECS	*Criança, Saúde, Saúde da Criança; População Negra; Disparidades nos Níveis de Saúde; Determinantes Sociais de Saúde; Grupos de Risco; Grupos com Ancestrais do Continente Africano; Populações Vulneráveis; Saúde das Minorias Étnicas; Quilombo; Quilombolas.*	Quilombolas; *Salud Infantil; Población Negra; Disparidades en los Niveles de Salud; Determinantes Sociales de la Salud; Grupos de Riesgo; Niños; Grupos Ancestrales del Continente Africano; Poblaciones Vulnerables; Salud de las Minorías Étnicas*; Quilombo.	Quilombola Communities; Child *Health*; Black Population; Disparities in Health Levels; Social Determinants of Health; Risk Groups; Vulnerable Groups; Children; Ancestral Groups from the African Continent; Vulnerable Populations; Health of Ethnic Minorities; *Quilombo*.
MESH	*Criança, Crianças, Saúde, Saúde da Criança, Determinantes Sociais da Saúde; Povos Negros; Pessoas Negras, Raça Negróide, Grupo de Ancestrais Continentais Africanos; População Negra.*	*Niño; Niños; Salud, Salud infantil; Determinantes Sociales de la Salud; Pueblos Negros; Gente Negra; Persona Negra; Personas Negras; Raza Negroide; Raza Negroide; Grupo de Ascendencia Continental Africana; Población Negra.*	Child, Children, Health, Child Health, Social Determinants of Health; Black Peoples; People, Black; Black Person; Black Persons; Persons, Black; Negroid Race; Race, Negroid; African Continental Ancestry Group; Black Population.
EMTREE	*‘Criança, Crianças, Saúde, Saúde Infantil; Pessoa Negra, Grupo De Ascendência Continental Africana; Homem Negro; Pessoas Negras, Determinantes Sociais Da Saúde; Nutrição Infantil; População Vulnerável; Grupo Étnico, Grupos Étnicos E Raciais’ OU ‘Minorias Étnicas E Raciais’ OU ‘Grupos Étnicos’ OU ‘Minorias Étnicas’, Territorialidade.*	*Niño; Niños, Salud, Salud Infantil; Persona Negra (Grupo de Ascendencia Continental Africana; Hombre Negro; Pueblo Negro); Determinantes Sociales de la Salud; Nutrición Infantil; Población Vulnerable; Grupo Étnico, Territorialidad.*	‘Children’, ‘Child, Health’, Child Health; Black Person (African Continental Ancestry Group; Black Man; Black People); Social Determinants of Health; Child Nutrition; Vulnerable Population; Ethnic Group, Territoriality.

The search took place in June and July 2023 in the *Literatura Latino-Aamericana e do Caribe em Ciências da Saúde* (LILACS) and *Base de Dados em Enfermagem* (BDENF) databases. Moreover, they were selected by online access using the Virtual Health Library (VHL) and the Google Scholar platform. Searches in the Scopus, MEDLINE, and Web of Science databases were carried out on the *Coordenação de Aperfeiçoamento de Pessoal de Nível Superior* (CAPES, Coordination for the Improvement of Higher Education Personnel) Journal Portal, accessed through the *Comunidade Acadêmica Federada* (CAFe, Federated Academic Community), linked to the *Universidade Federal do Maranhão* (UFMA).

The search strategy followed the criteria of primary articles that contained themes related to child health and belonging to the *quilombola* community. Gray literature and articles whose theme was not around the triad of health, child and *quilombola* were excluded. Articles that were not available in full, but that, based on reading the title and abstract, answered the guiding question, were accessed in other repositories, thus enabling subsequent readings. The next stage involves organizing the strings, and the Boolean operators OR and AND should be used to structure them. Chart 2 shows the search strategies applied to this study that were relevant to the topic under investigation.

**Chart 2:** Search results with full strings

Database	Full string	Number of records
LILACS	(*Quilombolas* OR *Quilombolas* OR *Quilombola* Communities) AND (*Crianças* OR *Niños* OR Children) AND (*Saúde da Criança* OR *Salud Infantil* OR Child Health).	745
BDENF	(*Quilombolas* OR *Quilombolas* OR *Quilombola* Communities) AND (*Crianças* OR *Niños* OR Children) AND (*Saúde da Criança* OR *Salud Infantil* OR Child Health).	75
MEDLINE	(*Crianças* OR *Niños* OR Children) AND (*Saúde das Minorias Étnicas* OR *Salud de las Minorías Étnicas* OR Health of Ethnic Minorities) AND (*População Negra* OR *Población Negra* OR Black Population).	39
MEDLINE	(African Continental Ancestry Group) AND (*Saúde* OR *Salud* OR Health) AND (*Criança* OR *Niño* OR Child).	942
Web of Science	(*Quilombolas* OR *Quilombolas* OR *Quilombola* Communities) AND (*Crianças* OR *Niños* OR Children) AND (*Saúde da Criança* OR *Salud Infantil* OR Child Health).	25
Scopus	(Social Determinants of Health) AND (Health Child OR Infant Health) AND (African Continental Ancestry Group).	93
	**Search platform**	
Google Scholar	(*Crianças* OR *Niños* OR Children) AND (*Saúde das Minorias Étnicas* OR *Salud de las Minorías Étnicas* OR Health of Ethnic Minorities) AND (*População Negra* OR *Población Negra* OR Black Population).	136

After searching the databases, one of the researchers exported the files to the Rayyan QCRI centralization tool, allowing the other reviewers to individually select eligible studies. In the event of disagreement, the decision was made by tiebreaker, based on the inclusion criteria, thus arriving at the final result of the study. This stage took place between July and September 2023. The process of systematically organizing studies for study follow-up and the analysis and construction of results used the Preferred Reporting Items for Systematic reviews and Meta-Analyses extension for Scoping Reviews (PRISMA-ScR)^(11)^.

## RESULTS

**Figure 1:**
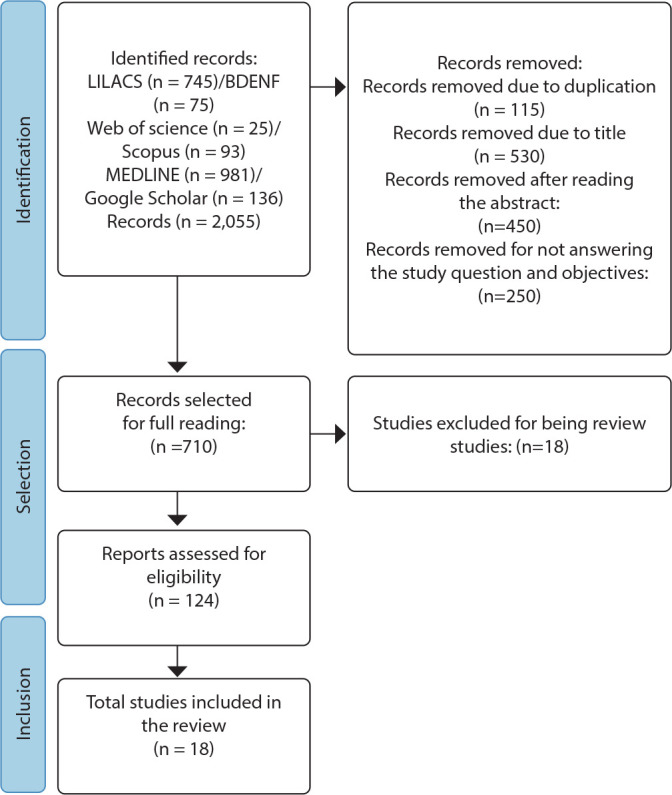
Adaptation of the PRISMA diagram referring to the study selection process, 2024

Eighteen articles were selected as relevant for this study, after careful analysis of 2055 studies, according to the PRISMA diagram^(12)^ (Figure 1).

**Figure 2:**
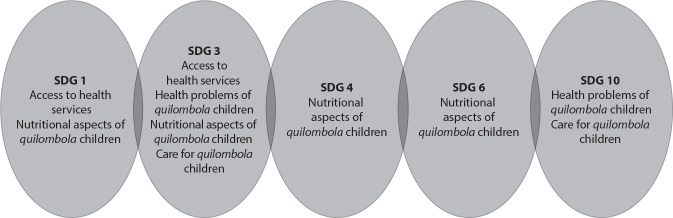
List of Sustainable Development Goals targets and the thematic axes of review studies, 2024

The studies that comprised the results of this research were organized in the following order: title; code assigned to studies S (study) from S1 to S18; journal; country of the journal; study design; objective; type and number of participants; and outcomes. They were then analyzed using simple descriptive statistics (relative and absolute)^(13)^, presented and discussed with theoretical basis in the literature, as described in Chart 3.

**Chart 3:** Characterization of selected studies, 2024

Study title/code	Journal/ country	Design	Objective	Sample of selected studies	Outcomes
**Access to health services**					
*Saúde materno-infantil em comunidades quilombolas no norte de Minas Gerais* ^(14)^ (S1)	*Cadernos de Saúde Coletiva*/ Brazil	Cross-sectional, descriptive study with a quantitative approach.	To describe maternal and child health indicators in *quilombola* communities in northern Minas Gerais.	Women and children (n=411; n= 234)	In relation to women, reproductive health points to restricted access to educational or family planning activities and a high percentage of children who were born with low birth weight.
*Atenção Primária e saúde materno-infantil: a percepção de cuidadores em uma comunidade rural quilombola* ^(15)^ (S2)	*Ciência & Saúde Coletiva*/ Brazil	Cross-sectional, descriptive study with a quantitative approach.	To assess the attributes of primary care, with a focus on child health, according to the perception of a *quilombola* community in northern Minas Gerais.	Child caregivers (n=76)	Only two primary care attributes presented satisfactory values: access, use and information system coordination. The worst scores were for family guidance and access-accessibility.
*Itinerário terapêutico em situações de urgência e emergência pediátrica em uma comunidade quilombola* ^(16)^ (S3)	*Ciência & Saúde Coletiva*/ Brazil	Non-randomized follow-up study with a qualitative approach.	To understand the therapeutic itinerary adopted by *quilombolas* in pediatric emergency care situations.	Mothers (n=12)	The care provided to children begins in the informal subsystem, and access to the formal subsystem was characterized as a pilgrimage through health services.
*Fatores relacionados à assiduidade de quilombolas às consultas de acompanhamento infantil* ^(17)^ (S4)	*Revista Brasileira de Enfermagem*/ Brazil	Cross-sectional study, with a qualitative approach.	To understand factors that interfere with the attendance of *quilombola* children at growth and development monitoring appointments.	Mothers (n=14)	The attendance of *quilombola* children at growth and development monitoring appointments is influenced by intrinsic factors, relevant to the mothers, and extrinsic factors, related to the health service.
**Health problems of *quilombola* children**					
*Mortalidade Infantil em Remanescentes de Quilombos do Município de Santarém - Pará, Brasil* ^(18)^ (S5)	*Saúde e Sociedade*/ Brazil	Survey-based study with a quantitative approach.	To assess the magnitude of infant mortality in six *quilombola* communities referenced for the municipality of Santarém-Pará.	*Quilombola* population (n= 2,197)	The results highlight profound inequalities, as mortality rates in *quilombola* communities are higher when compared to those in the country.
Factors associated with childhood anaemia in Afro-descendant communities in Alagoas, Brazil^(19)^ (S6)	Public Health Nutrition/ United Kingdom	Cross-sectional study, with a quantitative approach.	To investigate the factors associated with anemia in preschool children.	Mother-child dyad (n=428)	Factors associated with childhood anemia were male sex, age < 24 months, greater number of residents in the home (> 4), relatively taller mothers, and higher Body Mass Index z-score for age.
*Evolução da prevalência de anemia em crianças quilombolas, segundo dois inquéritos de base populacional em Alagoas, Brasil (2008-2018)* ^(20)^ (S7)	*Cadernos de Saúde Coletiva*/ Brazil	Case series study, with a quantitative approach.	To assess the evolution of the prevalence of anemia and hemoglobin levels, according to age group and sex, in *quilombola* children aged 6 to 59 months.	Children (2008, n = 950, and 2018, n = 426)	The prevalence of anemia declined from 2008 to 2018. Despite this, in younger age groups, the magnitude remained at levels above 40%, remaining a serious public health problem.
Concepts, Beliefs, and Traditional Treatment for Childhood Seizures in a Quilombola Community in Northeastern Brazil: Analysis by the Discourse of the Collective Speech^(21)^ (S8)	International Journal of Environmental Research and Public Health/ Switzerland	Case series study, quantitative approach.	To investigate which concepts, beliefs, possible stigmas and types of traditional treatments are used to manage acute and/or chronic childhood crises in a *quilombola* community.	Healers, prayers and midwives (n=19)	A total of 14 central ideas were found. The most prevalent was that convulsions are the most common type in children (50.0%), occurring due to fever (42.0%). In the community, they are treated and prevented with the use of plants (63.2%).
**Nutritional aspects of *quilombola* children**					
*(In) segurança alimentar de Comunidades Quilombolas do Tocantins* ^(22)^ (S9)	*Segurança Alimentar e Nutricional*/ Brazil	Case series study, with a quantitative approach.	To identify and analyze some determinants of food (in)security in families from 14 *quilombola* communities in the state of Tocantins.	Families (n=696)	Food insecurity (FI) was present in 85.1% of families. It was observed that 40.2% of households were made of adobe; only 25.4% had garbage collection; 31.3% had water supply; and 8.5% had sewage.
*Nutrição e saúde das crianças das comunidades remanescentes dos quilombos no Estado de Alagoas, Brasil* ^(23)^ (S10)	*Revista* *Panamericana de Salud Pública*/ United States	Cross-sectional study, with a quantitative approach.	To describe the nutritional and health conditions of children aged 6 to 59 months from 39 remaining *quilombo* communities in the state of Alagoas.	Children (n=973)	Most families belonged to class E (the poorest) and were assisted by the *Programa Bolsa Família* (Family Allowance). The heads of families had less than four years of education (75.9%), and 57.1% of households had more than five residents.
*Consumo alimentar e estado nutricional de pré-escolares das comunidades remanescentes dos quilombos do estado de Alagoas* ^(24)^ (S11)	*Revista Paulista de Pediatria*/ Brazil	Cross-sectional study, with a quantitative approach.	To assess food consumption and nutritional status of children from *quilombola* communities in Alagoas.	Children (n=724)	The prevalence of anemia, stunting and obesity was 48%, 9.7% and 6.0%, respectively. The children had a monotonous dietary pattern and a considerable prevalence of inadequate intake of zinc, folate, iron and vitamins A and C.
*Perspectivas de segurança alimentar e nutricional no Quilombo de Tijuaçu, Brasil: a produção da agricultura familiar para a alimentação escolar* ^(25)^ (S12)	*Interface - Comunicação, Saúde, Educação*/ Brazil	Case series study, with a qualitative approach.	To analyze the symbolic and social perceptions related to the supply of food produced by family farming to the *Programa Nacional de Alimentação Escolar* (PNAE, Brazilian National School Feeding Program).	Rural producers, members of the family nucleus, heads of households of both sexes, young people and students from *quilombos*, lunch ladies and community leaders (n=14)	The community conceives and values ​​the “natural” food of the land as a source of survival and local development.
*Insegurança alimentar em comunidades rurais no Nordeste brasileiro: faz diferença ser quilombola?* ^(26)^ (S13)	*Cadernos de Saúde Coletiva*/ Brazil	Cross-sectional study, with a quantitative approach.	To identify the prevalence of food insecurity in a rural area of northeastern Brazil and investigate the factors associated with this outcome, according to residence in *quilombola* and non-*quilombola* communities in the same coverage area.	*Quilombola* and non-*quilombola* families (n= 248; n= 294)	The prevalence of FI was high across the population, however, the *quilombola* communities, despite belonging to the same coverage area as the other communities, presented an even higher prevalence of FI.
*Desnutrição e fatores associados em crianças quilombolas menores de 60 meses em dois municípios do estado do Maranhão, Brasil* ^(27)^ (S14)	*Ciência & Saúde Coletiva*/ Brazil	Cross-sectional, descriptive, analytical study with a quantitative approach.	To assess the prevalence of malnutrition in children under 60 months living in remaining *quilombo* communities in two municipalities in the state of Maranhão and its associated factors.	Children (n=372)	Child malnutrition persists as a public health problem in vulnerable regions, and maternal factors such as maternal short stature may explain the short stature of children. Actions to address this nutritional deficiency are needed.
*Estado nutricional e fatores associados ao déficit estatural em crianças menores de cinco anos de comunidades remanescentes de quilombos do Nordeste brasileiro* ^(28)^ (S15)	*Cadernos de Saúde Coletiva*/ Brazil	Non-randomized follow-up study with a quantitative approach.	To assess the nutritional status and associated factors in *quilombola* children under five years of age living in titled *quilombola* communities in the Northeast region.	Inhabitants of *quilombola* communities (n= 40,548)	Stature deficit, indicative of chronic malnutrition in epidemiological studies, was the most prevalent condition among the children investigated, while the other nutritional conditions were considered to be of low magnitude.
*Correlação entre o estado nutricional e a prevalência de enteroparasitoses em crianças de uma comunidade quilombola da cidade de Caetés, Pernambuco* ^(29)^ (S16)	*O Mundo da Saúde*/ Brazil	Cross-sectional study, with a quantitative approach.	To verify whether there would be a correlation between intestinal parasites and the nutritional status of children in a *quilombola* community.	Children (n=155)	Approximately 70% of children were parasitized by protozoa. The presence of entamoebae is closely related to socio-environmental conditions, contamination of water and food consumed as well as precarious housing conditions.
*Avaliação do consumo alimentar e estado nutricional de crianças menores de dois anos de uma comunidade quilombola* ^(30)^ (S17)	*Arquivos de Ciências da Saúde da UNIPAR*/ Brazil	Quantitative approach study.	To assess food consumption and nutritional status of children aged 0 to 23 months who live in the *Quilombola* Community of Córrego dos Iús-CE.	Children (n=9)	The most important nutritional problems were high weight for age (44.4%), risk of overweight for height and obesity for height, presenting, respectively, overweight (11.1%) for Body Mass Index for age (11.1%).
**Care for *quilombola* children**					
*Práticas de cuidado em saúde com crianças quilombolas: percepção dos cuidadores* ^(31)^ (S18)	Escola de Enfermagem Anna Nery/ Brazil	Exploratory-descriptive study, with a qualitative approach.	To analyze, from caregivers’ perspective, the health care practices provided to *quilombola* children.	Female caregivers (n=18)	Caregivers related care to the prevention and treatment of diseases, lifestyle habits, access to health services and popular practices that value traditional medicine.

The research studies that were selected as relevant for this study were grouped into thematic axes, according to the similarity they presented, being the following: Access to health services; Health problems of *quilombola* children; Nutritional aspects of *quilombola* children; and Care for *quilombola* children.

Regarding the journals of the studies, the studies of *Ciência & Saúde Coletiva*, three, S2, S3, S14 (16.7%), and of *Cadernos de Saúde Pública*, three, S7, S13 and S15 (16.7%), predominated. Regarding the others, one study each: *Cadernos de Saúde Coletiva*, S1; *Revista Brasileira de Enfermagem*, S4; *Saúde e Sociedade*, S5; Public Health Nutrition, S6; International Journal of Environmental Research and Public Health, S8; *Segurança Alimentar e Nutricional*, S9; *Revista Panamericana de Salud Pública*, S10; *Revista Paulista de Pediatria*, S11; *Interface - Comunicação, Saúde, Educação*, S12; *O Mundo da Saúde*, S16; *Arquivos de Ciências da Saúde da Universidade Paranaense* (UNIPAR), S17; and *Escola de Enfermagem Anna Nery*, S18, all with (5.6%) each. In this regard, there are still few publications at an international level.

The axis that presents the majority of published studies is “Nutritional aspects of *quilombola* children”, nine in total, S9, S10, S11, S12, S13, S14, S15, S16 and S17 (50%), followed by “Access to health services”, four, S1, S2, S3 and S4 (22.2%), “Health problems of *quilombola* children”, four, S5, S6, S7 and S18 (22.2%), and “Care for *quilombola* children”, one, S17 (5.6%).

As for study objectives, in the “Access to health services” axis, S1 and S2 seek to describe how assistance occurs at the Primary Health Care level, while S3 and S4 aim to understand the path taken to this assistance and the factors related to the frequency of attendance. All studies in this axis indicate a direct relationship with at least five SDGs, namely: SDG 1: No poverty; SDG 3: Good health and well-being; SDG 4: Quality education; SDG 6: Clean water and sanitation; and SDG 10: Reduced inequalities^(3)^.

In the “Nutritional aspects of *quilombola* children” axis, the studies propose to assess food consumption (S9, S11 and S17), food security (S13 and S14), nutritional and socioeconomic conditions (S10 and S15) and the relationship between diet and the occurrence of diseases (S16). In this category, the close relationship between most studies is evident, especially with SDG 1: No poverty, SDG 3: Good health and well-being and SDG 6: Clean water and sanitation.

Concerning the results in the “Health problems of *quilombola* children” axis, studies showed a prevalence of anemia in children under 2 years of age associated with the greater number of residents in the household (S6), and the survey carried out between 2008 and 2018 (S7) showed that, despite the reduction in cases, it is still a serious health problem in this population group.

S5 found that the greater the socioeconomic inequalities, the higher the mortality rates in *quilombola* communities, and that in these communities these rates are above the national average. In this axis, the adequacy to SDG 1: No poverty, SDG 3: Good health and well-being and SDG 10: Reduced inequalities is clearly perceived.

S18, belonging to the axis “Care for *quilombola* children”, details, through an exploratory-descriptive study, caregivers’ perception in relation to the assistance offered by Primary Health Care, and its main results are the priority given by these caregivers to the practice of traditional medicine and its combination with the biomedical model offered by Primary Health Care. In this thematic axis, the relationship with SDG 3 and SDG 10 has been highlighted.

## DISCUSSION

According to the studies analyzed for this review, *quilombola* children can be directly associated with some of the SDGs. SDG 1 aims to eradicate poverty in all its forms, everywhere, as many *quilombola* children live in poverty with limited access to basic resources. SDG 3 addresses good health and well-being of all ages, considering possible disparities such as lack of access to basic care and poor sanitation conditions. It is evident that this objective reveals the challenges that especially affect *quilombola* children in Brazil in relation to access to health, the geographical barrier (distance from health institutions). In addition to infrastructure, resources, discrimination and, especially, nutritional aspects, it is observed that FI is a serious and prevalent problem. SDG 4 focuses on quality education for all, considering potential challenges due to structural and cultural inequalities. SDG 6 refers to safe water and sanitation for all, due to lack of household resources and lack of government investment. SDG 10 addresses reducing inequalities, as *quilombola* communities, including children, often face difficulties^(32,33)^.

The Strategy for Universal Access to Health and Universal Health Coverage recognizes the importance of quality health services that meet the health needs of people and communities. This involves paying special attention to diversity and to people and populations in vulnerable conditions. SDGs 1, 3 and 10, consistent with the studies presented in this review, are related to the promotion of quality health services. SDG 1 combats poverty; SDG 3 promotes health actions; and SDG 10 reduces economic, social and political inequalities^(34)^. Health problems of *quilombola* children related to economic, social and cultural factors, such as diseases, malnutrition and high mortality rates, have the potential to cause delays in the growth and development of these children.

In a study that deals with pediatric emergency situations, the demand is that health services have good conditions and a rapid response, in addition to ensuring access to them by the population. In the *quilombola* community, this access is hindered by a series of factors, such as geographic distance, unfavorable socioeconomic conditions and cultural barriers. Thus, it has a direct correlation with SDG 3, more specifically target 3.8, as it highlights the importance of ensuring full and easy access to health services^(3)^.

SDG 3 has as one of its goals until 2030: “Reduce premature mortality from non-communicable diseases (NCDs) by one third through prevention and treatment and promote mental health and well-being”^(33)^. Although anemia is not a non-communicable disease, reducing the prevalence of anemia in *quilombola* children in Alagoas, Brazil, can be considered an important step towards achieving SDG 3, as anemia persists as a public health problem, especially among children aged 6 to 24 months.

Access to the communities is via local roads, some of which are difficult to navigate, especially during the rainy season, when potholes and mud become more frequent. In this context, it can be said that the quality of roads is directly linked to public health, education and leisure issues, so their maintenance should always be on the development agenda of municipal managers^(35)^.

Nursing plays an important role in SDG 3, which aims to ensure healthy lives and promote well-being for all at all ages. In the multidisciplinary team, nursing is essential for promoting health and preventing diseases, contributing to the achievement of goals related to infant mortality, maternal health and disease control. This also involves reducing child malnutrition, which directly affects the well-being, longevity and quality of life of the world’s population, especially *quilombola* children, who suffer from a lack of access to basic resources, such as drinking water, sanitation and food^(36)^.

Furthermore, health professionals, especially nurses, as an integral part of the complex global socio-environmental system, are called upon to contribute with all possible efforts to achieve the SDGs^(8)^. Nursing can play a role in valuing the traditional knowledge and practices of *quilombola* communities, strengthening primary care. With regard to *quilombola* children, it has an intrinsic relationship with SDG 3, promoting health and well-being, SDG 4, promoting quality education, in this case, health education, SDG 6, ensuring the availability and sustainable management of water and sanitation for all, and SDG 10, reducing inequalities related to lack of access to health services, for instance^(37)^.

In another study that deals with child malnutrition, a correlation with this goal is also found, in addition to pointing out other aggravating factors, which are the prevalence of chronic malnutrition and obesity. The *Vigilância da Segurança Alimentar e Nutricional* (II VIGISAN, Food and Nutrition Security Surveillance) describes the food security conditions and the FI levels of households in the Federative Units of Brazil. The analyses cover a selection of 12,745 households, located in urban and rural areas. There is a proportion of moderate and severe FI, above 30.0%, in households with children under 10 years old^(38)^.

Preventable infant mortality and social inequalities are major challenges facing lowand middle-income countries (LMICs). Differences in morbidity and mortality have been widely discussed across groups, such as ethnicity, sex, place of residence and socioeconomic status^(39)^. Although the relationship with child malnutrition is not direct, malnutrition is one of the main factors contributing to child mortality in children under 5 years of age. Therefore, reducing malnutrition is an important step towards achieving SDG 3^(40)^.

The relationship with SDG 3, present in studies that address FI in *quilombola* communities, is that hunger and FI are associated with poor academic and behavioral performance in children, and are responsible for more than 50% of all deaths in children under 5 years of age. To achieve the SDG of abolishing hunger by 2030, it is necessary to implement adequate public health methods and policies that guarantee access to nutritious food^(40)^.

Ensuring the availability and sustainable management of water and sanitation for all is the central goal of SDG 6, and is present in studies that address FI in *quilombola* children, since, in many of these communities, only a quarter of households had garbage collection, and a small number had water supply and sanitation. Hence, they oppose indicator 6.1: by 2030, achieve universal and equitable access to safe and secure drinking water for all^(41)^.

A similar relationship occurs in a study that verified the occurrence of enteroparasitosis in *quilombola* children, demonstrating its close relationship with socio-environmental conditions, contamination of water and food consumed. Socioeconomic and sanitation conditions are consistent with other research. Historical processes of segregation and discrimination have left these communities with inherited disadvantages. These findings are in line with the need to achieve SDG target 6.2, achieving access to adequate and equitable sanitation and hygiene for all, and ending open defecation, especially for those in vulnerable situations^(7,41)^.

From the period of slavery to the present day, estimates of the material living and health conditions of black people in relation to white people are worse, characterized by worse sanitary and health infrastructure in the places where they live, lack of access to equipment and social institutions, such as schools, income and health services. With the aim of reducing inequality within and between countries, goal 10, in one of its indicators, aims to empower and promote the social, economic and political inclusion of all, regardless of age, gender, disability, race, ethnicity, origin, religion, economic or other condition by 2030^(42)^.

Contributing to achieving the SDGs, Primary Health Care is considered the best way to ensure sustainable improvements in health, society and environmental outcomes, when supported by strong public policies aligned with the economic, political and social spheres. The SDG report highlights that, although progress has been made in some areas, there are still major challenges to be overcome. These challenges and commitments are interrelated and require integrated solutions. It is imperative to take a holistic view of the 2030 Agenda and identify the most affected areas to implement targeted interventions. In this regard, valuable opportunities may arise to accelerate progress by analyzing the interrelationships between the goals^(43,44)^.

Faced with this scenario of injustice, the *quilombolas* resist and seek to maintain their solidarity networks, their traditions and their relationship with nature. In this way, these historical, social and cultural characteristics are related to *quilombola* population’s illness and health processes^(45,46)^. Furthermore, the growing human population has demanded the accelerated development of infrastructure, such as housing, health, quality roads, education, communication and related services. Traditional lifestyles seen in *quilombola* communities are gradually being replaced by technology-driven living standards^(47)^.

### Study limitations

The limited number of international publications related to *quilombola* children may have limited the discussions presented in the review. Furthermore, even in the national literature, most of studies found do not refer specifically to this age group.

### Contributions to health, nursing or public policy

The relevance of this study for health, especially for nursing, lies in the fact that it presents a possible indication of the great possibility of developing more research with the *quilombola* community. Nursing, especially with its extensive experience in ethnography, has all the necessary tools to develop studies that will contribute to the advancement of public policies aimed at this population, including children.

## CONCLUSIONS

It is concluded that this study provided a mapping of extremely important studies on *quilombola* children’s health and their relationship with the SDGs. This clarified the understanding of which topics related to *quilombola* children are being addressed and debated. Through this survey of national and international databases and the use of various combinations of descriptors, it was possible to infer that the literature produced revolves around topics more closely related to the axes defined in this article and that have a direct relationship with the SDGs.
