# Supplementary material for: Health of quilombola children as a challenge for the Sustainable Development Goals: a scoping review
Source: Rev Bras Enferm. 2025 Jan 10;77(Suppl 2):e20240106. doi: 10.1590/0034-7167-2024-0106 (PMC11726813; doi:10.1590/0034-7167-2024-0106)
Supplement: Translation

# Translation: Saúde da criança quilombola como desafio para os Objetivos de Desenvolvimento Sustentável: revisão de escopo

**DOI:** 10.1590/0034-7167-2024-0106pt

**Authors:** Emanuella Pereira de Lacerda, Sara Fiterman Lima, Bruno Luciano Carneiro Alves de Oliveira

**Affiliations:** I Universidade Federal do Maranhão. São Luís, Maranhão, Brasil

**Keywords:** Criança, Saúde da Criança, População Negra, Quilombo, Quilombolas

## Abstract

**Objetivo:**

mapear a literatura sobre a saúde de crianças quilombolas e a relação com os Objetivos de Desenvolvimento Sustentável.

**Método:**

revisão de escopo seguiu o protocolo do JBI e o *Preferred Reporting Items for Systematic reviews and Meta-Analyses extension for Scoping Reviews*. As buscas foram nas bases LILACS, BDENF, *Web of Science, Scopus,* MEDLINE e na plataforma *Google* Acadêmico. O protocolo de pesquisa foi registrado no *Open Science Framework*.

**Resultados:**

foram selecionados como relevantes para este estudo 18 artigos de 2.055 estudos. Os artigos foram agrupados em quatro eixos: Acesso aos serviços de saúde; Aspectos nutricionais da criança quilombola; Agravos à saúde da criança quilombola; e Cuidado à criança quilombola. Observou-se a relação desses com Objetivos 1, 3, 4, 6 e 10.

**Considerações finais:**

o estudo proporcionou um mapeamento de suma importância sobre a temática da saúde de crianças quilombolas e as temáticas relacionadas aos Objetivos de Desenvolvimento Sustentável.

## INTRODUÇÃO

Os Objetivos de Desenvolvimento Sustentável (ODS), lançados pela primeira vez em 2015, durante a Assembleia Geral da Organização das Nações Unidas (ONU), buscam acabar com a pobreza, proteger o planeta e garantir que todas as pessoas desfrutem de paz e prosperidade. Os ODS possuem quatro principais dimensões (social, ambiental, econômica e institucional) e 17 objetivos universais, os quais são relevantes para as comunidades quilombolas, que enfrentam desigualdades sociais, econômicas e ambientais^(1,2)^.

Relativamente à criança quilombola, pode-se destacar uma relação direta com os seguintes ODS: o ODS 1, de erradicação da pobreza em todas as suas formas, em todos os lugares, pois muitas vivem em situações de pobreza e com acesso limitado a recursos básicos; e o ODS 3, que busca garantir uma vida saudável e promover o bem-estar para todos, em todas as idades, considerando possíveis disparidades como falta de acesso à atenção básica e precárias condições de saneamento.

Considerando possíveis disparidades, como falta de acesso à atenção básica e precárias condições de saneamento, destacam-se os ODS: ODS 4, que foca em educação de qualidade para todos, considerando potenciais desafios por desigualdades estruturais e culturais; o ODS 6, que trata de assegurar a disponibilidade e gestão sustentável da água e saneamento para todas e todos; e o ODS 10, cujo objetivo é reduzir as desigualdades econômicas, sociais e políticas, pois as comunidades quilombolas, incluindo as crianças, frequentemente enfrentam altos níveis de desigualdade social e econômica ^(3,4)^.

Recentemente, o Projeto Quilombos e Educação, desenvolvido pela Coordenação Nacional de Articulação das Comunidades Negras Rurais Quilombolas (CONAQ)^(5)^, vem pautando as necessidades sociais e de saúde de crianças quilombolas no Brasil, visto que o país tem aproximadamente 151 mil alunos matriculados em 1.253 escolas localizadas em comunidades quilombolas, o que revela uma população considerável^(6)^. Apesar disso, as condições de vida e saúde das crianças quilombolas ainda recebem pouca atenção diante da magnitude dessa temática, e os estudos disponíveis revelam problemas de saúde pública significativos, como qualidade de vida, problemas de saneamento básico e dificuldades de acesso a serviços sociais e de saúde, especialmente entre os extremos de idade, como idosos e crianças^(7)^. Embora tais resultados tenham clara relação com os ODS, muitos desses estudos não estabelecem essa relação.

É importante destacar a interface com a enfermagem, visto que existe uma estreita ligação dessa área com os ODS, revelada a partir do dia a dia de trabalho, em atuação comprometida com a promoção da saúde e o bem-estar dos que estão sob seus cuidados, em todas as faixas etárias e em diversos contextos socioambientais, desempenhando papel vital e transformando as metas globais em ações tangíveis e na promoção de um mundo mais saudável, equitativo e sustentável^(8)^.

Neste contexto, entende-se que a presente revisão de escopo se torna relevante, uma vez que permite, para além do levantamento das evidências científicas, evidenciar a saúde das crianças quilombolas e sua correlação com os ODS, principalmente o ODS 3, que trata da saúde e do bem-estar. Dessa forma, permite um olhar diferenciado sobre o tema e uma reflexão sobre a inclusão dessa população em agendas que visem mudanças para as comunidades específicas, como as crianças quilombolas.

## OBJETIVO

Mapear a literatura sobre a saúde de crianças quilombolas e sua relação com os ODS.

## MÉTODOS

### Aspectos éticos

Por tratar-se de estudo de revisão de escopo, o presente estudo dispensa a apreciação por Comitê de Ética em Pesquisa.

### Tipo de estudo

Trata-se de revisão de escopo, que tem o intuito de identificar na literatura evidências científicas sobre a saúde da criança quilombola e, principalmente, sua relação com os ODS, em especial o ODS 3: Saúde e bem-estar.

### Procedimento metodológico

Para revisão, o percurso metodológico seguiu o protocolo do JBI^(9)^, que é um guia para revisão de escopo. A instrumentalização metodológica ocorreu por meio de uma organização elaborada em um editor de planilhas, nomeadamente *Microsoft Office Excel^®^,* com a execução de quatro passos, os quais foram realizados nos meses de junho e julho de 2023, sendo eles: 1) identificação da questão de pesquisa; 2) identificação dos estudos relevantes; 3) seleção dos estudos; 4) análise dos estudos; e 5) agrupamento, síntese e apresentação dos dados.

O protocolo de pesquisa com definição de critérios, transparência e replicação foi registrado na plataforma *Open Science Framework*, sob o identificador 10.17605/OSF.IO/37SU5^(10)^. A pergunta do estudo foi formulada de acordo com o acrônimo *Participants, Concept, Context* (PCC). Para este estudo, o P refere-se a crianças quilombolas, C, à saúde, e C, a mundo. As questões do estudo foram: quais temas relacionados à saúde da criança quilombola estão disponíveis na literatura? Quais aspectos da saúde da criança quilombola estão à luz dos ODS? A etapa seguinte foi realizada com a pesquisa nas bases de dados, por meio de acesso institucional. O terceiro passo foi a seleção de um vocabulário controlado a partir dos Descritores em Ciências da Saúde (DeCS), os *Medical Subject Headings Section* (MeSH) e EMTREE, conforme mostra o Quadro 1.

**Quadro 1:** Localização dos termos para o estudo

	Termo em português	Termo em espanhol	Termo em inglês
DeCS	Criança, Saúde, Saúde da Criança; População Negra; Disparidades nos Níveis de Saúde; Determinantes Sociais de Saúde; Grupos de Risco; Grupos com Ancestrais do Continente Africano; Populações Vulneráveis; Saúde das Minorias Étnicas; Quilombo; Quilombolas.	Quilombolas; *Salud Infantil; Población Negra; Disparidades en los Niveles de Salud; Determinantes Sociales de la Salud; Grupos de Riesgo; Niños; Grupos Ancestrales del Continente Africano; Poblaciones Vulnerables; Salud de las Minorías Étnicas*; Quilombo.	Quilombola *Communities; Child Health; Black Population; Disparities in Health Levels; Social Determinants of Health; Risk Groups; Vulnerable Groups; Children; Ancestral Groups from the African Continent; Vulnerable Populations; Health of Ethnic Minorities*; Quilombo.
MESH	Criança, Crianças, Saúde, Saúde da Criança, Determinantes Sociais da Saúde; Povos Negros; Pessoas Negras, Raça Negróide, Grupo de Ancestrais Continentais Africanos; População Negra.	*Niño; Niños; Salud, Salud infantil; Determinantes Sociales de la Salud; Pueblos Negros; Gente Negra; Persona Negra; Personas Negras; Raza Negroide; Raza Negroide; Grupo de Ascendencia Continental Africana; Población Negra.*	*Child, Children, Health, Child Health, Social Determinants of Health; Black Peoples; People, Black; Black Person; Black Persons; Persons, Black; Negroid Race; Race, Negroid; African Continental Ancestry Group; Black Population.*
EMTREE	‘Criança, Crianças, Saúde, Saúde Infantil; Pessoa Negra, Grupo De Ascendência Continental Africana; Homem Negro; Pessoas Negras, Determinantes Sociais Da Saúde; Nutrição Infantil; População Vulnerável; Grupo Étnico, Grupos Étnicos E Raciais’ OU ‘Minorias Étnicas E Raciais’ OU ‘Grupos Étnicos’ OU ‘Minorias Étnicas’, Territorialidade.	*Niño; Niños, Salud, Salud Infantil; Persona Negra (Grupo de Ascendencia Continental Africana; Hombre Negro; Pueblo Negro); Determinantes Sociales de la Salud; Nutrición Infantil; Población Vulnerable; Grupo Étnico, Territorialidad.*	*‘Children’, ‘Child, Health’, Child Health; Black Person (African Continental Ancestry Group; Black Man; Black People); Social Determinants of Health; Child Nutrition; Vulnerable Population; Ethnic Group, Territoriality.*

A busca ocorreu nos meses de junho e julho de 2023 nas bases de dados Literatura Latino-Americana e do Caribe em Ciências da Saúde (LILACS) e Base de Dados em Enfermagem (BDENF). Além disso, foram selecionados por acesso *online* utilizando a Biblioteca Virtual em Saúde (BVS) e a plataforma do *Google* Acadêmico. As buscas nas bases de dados Scopus, MEDLINE e *Web of Science* foram realizadas no Portal de Periódicos da Coordenação de Aperfeiçoamento de Pessoal de Nível Superior (CAPES), acesso por meio da Comunidade Acadêmica Federada (CAFe), vinculado à Universidade Federal do Maranhão (UFMA).

A estratégia de busca seguiu os critérios de artigos primários que continham temas relacionados à saúde da criança e pertencentes à comunidade quilombola. Excluíram-se a literatura cinzenta e artigos cujo tema não estivesse em torno da tríade saúde, criança e quilombola. Os artigos que não estavam disponíveis na íntegra, mas que, a partir da leitura do título e do resumo, respondiam à questão norteadora, foram acessados em outros repositórios, possibilitando, assim, leituras posteriores. A etapa subsequente refere-se à organização dos *strings*, e para sua estruturação, devem ser utilizados os operadores booleanos *OR e AND*. O Quadro 2 mostra as estratégias de busca aplicadas a este estudo e que foram relevantes para o tema investigado.

**Quadro 2:** Resultados das buscas com os *strings* completos

Base de dados	*String* completo	Número de registros
LILACS	(Quilombolas *OR* Quilombolas *OR* Quilombola *Communities*) *AND* (Crianças *OR Niños OR Children*) *AND* (Saúde da Criança *OR Salud Infantil OR Child Health*).	745
BDENF	(Quilombolas *OR* Quilombolas *OR* Quilombola *Communities*) *AND* (Crianças *OR Niños OR Children*) *AND* (Saúde da Criança *OR Salud Infantil OR Child Health*).	75
MEDLINE	(Crianças *OR Niños OR Children*) *AND* (Saúde das Minorias Étnicas *OR Salud de las Minorías Étnicas OR Health of Ethnic Minorities*) *AND* (População Negra *OR Población Negra OR Black Population*).	39
MEDLINE	(*African Continental Ancestry Group*) *AND* (Saúde *OR Salud OR Health*) *AND* (Criança *OR Niño OR Child*).	942
*Web of Science*	(Quilombolas *OR* Quilombolas *OR* Quilombola *Communities*) *AND* (Crianças *OR Niños OR Children*) *AND* (Saúde da Criança *OR Salud Infantil OR Child Health*).	25
Scopus	(*Social Determinants of Health*) *AND* (*Health Child OR Infant Health*) *AND* (*African Continental Ancestry Group*).	93
	**Plataforma de busca**	
*Google* Acadêmico	(Crianças *OR Niños OR Children*) *AND* (Saúde das Minorias Étnicas *OR Salud de las Minorías Étnicas OR Health of Ethnic Minorities*) *AND* (População Negra *OR Población Negra OR Black Population*).	136

Após as buscas nas bases de dados, um dos pesquisadores procedeu à exportação dos arquivos para a ferramenta de centralização *Rayyan QCRI,* possibilitando que os demais revisores realizassem a seleção individual dos estudos elegíveis, e, no caso de divergência, a decisão era tomada por desempate, com base nos critérios de inclusão, assim, chegando-se ao resultado final do estudo. Essa etapa ocorreu entre julho e setembro de 2023. O processo de organização sistemática dos estudos para seguimento do estudo e a análise e construção dos resultados utilizaram o *Preferred Reporting Items for Systematic reviews and Meta-Analyses extension for Scoping Reviews* (PRISMA-ScR)^(11)^.

## RESULTADOS

Foram selecionados como relevantes para este estudo 18 artigos, após criteriosa análise de 2.055 estudos, conforme diagrama PRISMA^(12)^ (Figura 1).

**Figura 1:** Adaptação do diagrama PRISMA referente ao processo de seleção dos estudos, 2024

**Figura 2:** Relação das metas dos Objetivos de Desenvolvimento Sustentável com os eixos temáticos dos estudos da revisão, 2024

Os estudos que compuseram os resultados desta pesquisa foram organizados na seguinte ordem: título; código atribuído aos estudos E (estudo) de E1 a E18; periódico; país do periódico; tipo de estudo; objetivo; tipo e número de participantes; e desfechos. Em seguida, foram analisados por estatística descritiva simples (relativa e absoluta)^(13)^, apresentados e discutidos com embasamento teórico na literatura, conforme descrito no Quadro 3.

**Quadro 3:** Caracterização dos estudos selecionados, 2024

Título/código do estudo	Periódico/ país	Delineamento	Objetivo	Amostra dos estudos selecionados	Desfechos
**Acesso aos serviços de saúde**					
Saúde materno-infantil em comunidades quilombolas no norte de Minas Gerais^(14)^ (E1).	Cadernos de Saúde Coletiva/ Brasil.	Estudo transversal, descritivo e de abordagem quantitativa.	Descrever indicadores de saúde materno-infantil em comunidades quilombolas ao norte de Minas Gerais.	Mulheres e crianças (n=411; n= 234).	Em relação às mulheres, a saúde reprodutiva aponta para um acesso restrito às atividades educativas ou de planejamento familiar e porcentual elevado de crianças que nasceram de baixo peso.
Atenção Primária e saúde materno-infantil: a percepção de cuidadores em uma comunidade rural quilombola^(15)^ (E2).	Ciência & Saúde Coletiva/ Brasil	Estudo transversal, descritivo, de abordagem quantitativa.	Avaliar os atributos da atenção primária, com enfoque na saúde infantil, segundo a percepção de uma comunidade quilombola no norte de Minas Gerais.	Cuidadores das crianças (n=76)	Apenas dois atributos da atenção primária apresentaram valores satisfatórios: acesso, utilização e coordenação-sistema de informação. Os piores escores foram para orientação familiar e acesso-acessibilidade.
Itinerário terapêutico em situações de urgência e emergência pediátrica em uma comunidade quilombola^(16)^ (E3)	Ciência & Saúde Coletiva/ Brasil	Estudo de acompanhamento não aleatório, de abordagem qualitativa.	Conhecer o itinerário terapêutico adotado por quilombolas em situações de urgência/emergência pediátrica.	Mães (n=12)	Os cuidados prestados à criança se iniciam no subsistema informal, e o acesso ao subsistema formal foi caracterizado como uma peregrinação pelos serviços de saúde.
Fatores relacionados à assiduidade de quilombolas às consultas de acompanhamento infantil^(17)^ (E4)	Revista Brasileira de Enfermagem/ Brasil	Estudo transversal, de abordagem qualitativa.	Apreender fatores que interferem na assiduidade de crianças quilombolas às consultas de acompanhamento do crescimento e desenvolvimento.	Mães (n=14)	A assiduidade de crianças quilombolas às consultas de acompanhamento do crescimento e desenvolvimento sofre interferência de fatores intrínsecos, pertinentes às mães, e extrínsecos, relacionados ao serviço de saúde.
**Agravos à saúde da criança quilombola**					
Mortalidade Infantil em Remanescentes de Quilombos do Município de Santarém - Pará, Brasil^(18)^ (E5)	Saúde e Sociedade/ Brasil	Estudo baseado em inquéritos, de abordagem quantitativa.	Avaliar a magnitude da mortalidade infantil em seis comunidades quilombolas referenciados para o município de Santarém, Pará.	População quilombola (n= 2.197)	Os resultados evidenciam profundas desigualdades, na medida em que as taxas de mortalidade das comunidades quilombolas são maiores quando comparadas com as do país.
*Factors associated with childhood anaemia in Afro-descendant communities in Alagoas, Brazil* ^(19)^ (E6)	*Public Health Nutrition*/ Reino Unido	Estudo transversal, de abordagem quantitativa.	Investigar os fatores associados à anemia em crianças pré-escolares.	Pares de mãecriança (n=428)	Os fatores associados à anemia infantil foram sexo masculino, idade < 24 meses, maior número de residentes em casa (> 4), mães relativamente mais altas e maior z-score de Índice de Massa Corporal para a idade.
Evolução da prevalência de anemia em crianças quilombolas, segundo dois inquéritos de base populacional em Alagoas, Brasil (2008-2018)^(20)^ (E7)	Cadernos de Saúde Pública/ Brasil	Estudo de série de casos, de abordagem quantitativa.	Avaliar a evolução da prevalência de anemia e dos níveis de hemoglobina, segundo faixa etária e sexo, em crianças quilombolas de 6 a 59 meses.	Crianças (2008, n = 950, e 2018, n = 426)	A prevalência de anemia apresentou um declínio de 2008 a 2018. Apesar disso, nas faixas etárias mais jovens, a magnitude permaneceu em patamares superiores a 40%, mantendo-se na condição de grave problema de saúde pública.
*Concepts, Beliefs, and Traditional Treatment for Childhood Seizures in a Quilombola Community in Northeastern Brazil: Analysis by the Discourse of the Collective Speech^(21)^* (E8)	*International Journal of Environmental Research and Public Health*/ Suíça	Estudo de série de casos, abordagem quantitativa.	Investigar quais conceitos, crenças, possíveis estigmas e tipos de tratamentos tradicionais utilizados para o manejo de crises agudas e/ou crônicas na infância crônicas em uma comunidade quilombola.	Curandeiras, rezadoras e parteiras (n=19)	Encontrou-se um total de 14 ideias centrais. A mais prevalente foi que a convulsão é o tipo mais comum em crianças (50,0%), ocorrendo por causa da febre (42,0%). Na comunidade, é tratada e prevenida com o uso de plantas (63,2%).
**Aspectos nutricionais da criança quilombola**					
(In) segurança alimentar de Comunidades Quilombolas do Tocantins^(22)^ (E9)	Segurança Alimentar e Nutricional/ Brasil	Estudo de série de casos, de abordagem quantitativa	Identificar e analisar alguns determinantes de (in)segurança alimentar nas famílias de 14 comunidades quilombolas do estado do Tocantins.	Famílias (n=696)	A insegurança alimentar (IA) esteve presente em 85,1% das famílias. Observou-se que 40,2% domicílios eram de adobe; apenas 25,4% possuíam coleta de lixo; 31,3% dispunham de abastecimento de água; e 8,5% dispunham de esgotamento sanitário.
Nutrição e saúde das crianças das comunidades remanescentes dos quilombos no Estado de Alagoas, Brasil^(23)^ (E10)	*Revista* *Panamericana de Salud Pública*/ Estados Unidos	Estudo transversal, de abordagem quantitativa	Descrever as condições de nutrição e saúde das crianças de 6 a 59 meses de 39 comunidades remanescentes dos quilombos no estado de Alagoas.	Crianças (n=973)	A maioria das famílias pertencia à classe E (a mais pobre), e era assistida pelo Programa Bolsa Família. Os chefes de família apresentavam escolaridade menor que quatro anos de estudo (75,9%), e 57,1% dos domicílios tinham mais de cinco moradores.
Consumo alimentar e estado nutricional de pré-escolares das comunidades remanescentes dos quilombos do estado de Alagoas^(24)^ (E11)	Revista Paulista de Pediatria/ Brasil	Estudo transversal, de abordagem quantitativa	Avaliar o consumo alimentar e o estado nutricional das crianças das comunidades quilombolas de Alagoas.	Crianças (n=724)	As prevalências de anemia, déficit estatural e obesidade foram, respectivamente, 48%, 9,7% e 6,0%. As crianças tinham um padrão alimentar monótono e uma considerável prevalência de inadequação na ingestão de zinco, folato, ferro e vitaminas A e C.
Perspectivas de segurança alimentar e nutricional no Quilombo de Tijuaçu, Brasil: a produção da agricultura familiar para a alimentação escolar^(25)^ (E12)	Interface - Comunicação, Saúde, Educação/ Brasil	Estudo de série de casos, de abordagem qualitativa	Analisar as percepções simbólicas e sociais relacionadas à oferta de alimentos de produção da agricultura familiar ao Programa Nacional de Alimentação Escolar (PNAE).	Produtores rurais, membros do núcleo familiar, chefes de família de ambos os sexos, jovens e estudantes quilombolas, merendeiras e lideranças comunitárias (n=14)	A comunidade concebe e valoriza o alimento “natural” da terra como fonte de sobrevivência e desenvolvimento local.
Insegurança alimentar em comunidades rurais no Nordeste brasileiro: faz diferença ser quilombola?^(26)^ (E13)	Cadernos de Saúde Pública/ Brasil	Estudo transversal, de abordagem quantitativa.	Identificar a prevalência de IA em uma zona rural do nordeste do Brasil e investigar os fatores associados a esse desfecho, de acordo com a residência em comunidades quilombolas e não quilombolas de uma mesma área de abrangência.	Famílias quilombolas e não quilombolas (n= 248; n= 294)	A prevalência de IA foi elevada em toda a população, no entanto as comunidades quilombolas, apesar de pertencerem à mesma área de abrangência das outras comunidades, apresentaram uma prevalência ainda maior de IA.
Desnutrição e fatores associados em crianças quilombolas menores de 60 meses em dois municípios do estado do Maranhão, Brasil^(27)^ (E14)	Ciência & Saúde Coletiva/ Brasil	Estudo transversal, descritivo, analítico, de abordagem quantitativa	Avaliar a prevalência de desnutrição em crianças menores de 60 meses residentes em comunidades remanescentes de quilombos de dois municípios do estado do Maranhão e seus fatores associados.	Crianças (n=372)	A desnutrição infantil persiste como problema de saúde pública em regiões vulneráveis, e os fatores maternos como a baixa estatura materna podem explicar a baixa estatura dos filhos. Aponta-se para a necessidade de ações de enfrentamento deste desvio nutricional.
Estado nutricional e fatores associados ao déficit estatural em crianças menores de cinco anos de comunidades remanescentes de quilombos do Nordeste brasileiro^(28)^ (E15)	Cadernos de Saúde Pública/ Brasil	Estudo de acompanhamento não aleatório, de abordagem quantitativa	Avaliar o estado nutricional e fatores associados em crianças quilombolas menores de cinco anos residentes em comunidades quilombolas tituladas na região Nordeste.	Habitantes de comunidades quilombolas (n= 40.548)	O déficit estatural, indicativo de desnutrição crônica em estudos epidemiológicos, foi à condição mais prevalente entre as crianças investigadas, ao passo que as demais condições nutricionais foram consideradas de baixa magnitude.
Correlação entre o estado nutricional e a prevalência de enteroparasitoses em crianças de uma comunidade quilombola da cidade de Caetés, Pernambuco^(29)^ (E16)	O Mundo da Saúde/ Brasil	Estudo transversal, de abordagem quantitativa.	Verificar se haveria correlação entre as parasitoses intestinais e o estado nutricional de crianças em uma comunidade quilombola.	Crianças (n=155)	Cerca de 70% das crianças foram parasitadas por protozoários. A presença das entamoebas está intimamente relacionada às condições socioambientais, contaminação da água e alimentos consumidos, além de precárias condições de moradia.
Avaliação do consumo alimentar e estado nutricional de crianças menores de dois anos de uma comunidade quilombola^(30)^ (E17)	Arquivos de Ciências da Saúde da UNIPAR/ Brasil	Estudo de abordagem quantitativa	Avaliar o consumo alimentar e o estado nutricional de crianças de 0 a 23 meses que residem na Comunidade Quilombola de Córrego dos Iús, Ceará.	Crianças (n=9)	Os agravos nutricionais mais importantes foram peso elevado para idade (44,4%), risco de sobrepeso para estatura e obesidade para estatura, apresentando, respectivamente, sobrepeso (11,1%) para Índice de Massa Corporal por idade (11,1%).
**Cuidado com a criança quilombola**					
Práticas de cuidado em saúde com crianças quilombolas: percepção dos cuidadores^(31)^ (E18)	Escola de Enfermagem Anna Nery/ Brasil	Estudo exploratório -descritivo, de abordagem qualitativa	Analisar, na percepção dos cuidadores, as práticas de cuidado em saúde prestadas às crianças quilombolas.	Cuidadores do sexo feminino (n=18)	Os cuidadores relacionaram o cuidado à prevenção e ao tratamento de doenças, aos hábitos de vida, ao acesso aos serviços de saúde e às práticas populares que valorizam a medicina tradicional.

As pesquisas que foram selecionadas como relevantes para este estudo foram agrupadas em eixos temáticos, de acordo com a similaridade que apresentaram, sendo os seguintes: Acesso aos serviços de saúde; Agravos à saúde da criança quilombola; Aspectos nutricionais da criança quilombola; e Cuidado à criança quilombola.

Em relação aos periódicos dos estudos, predominaram as publicações da Ciência & Saúde Coletiva, três, E2, E3, E14 (16,7%), e dos Cadernos de Saúde Pública, três, E7, E13 e E15 (16,7%). Em relação aos demais, um estudo cada: Cadernos de Saúde Coletiva, E1; Revista Brasileira de Enfermagem, E4; Saúde e Sociedade, E5; *Public Health Nutrition*, E6; *International Journal of Environmental Research and Public Health*, E8; Segurança Alimentar e Nutricional, E9; *Revista Panamericana de Salud Pública*, E10; Revista Paulista de Pediatria, E11; Interface - Comunicação, Saúde, Educação, E12; O Mundo da Saúde, E16; Arquivos de Ciências da Saúde da Universidade Paranaense (UNIPAR), E17; e Escola de Enfermagem Anna Nery, E18, todos com (5,6%) cada. Nesse quesito, percebem-se ainda poucas publicações a nível internacional.

O eixo que apresenta a maioria dos estudos publicados é “Aspectos nutricionais da criança quilombola”, nove ao todo, E9, E10, E11, E12, E13, E14, E15, E16 e E17 (50%), seguido dos eixos “Acesso aos serviços de saúde”, quatro, E1, E2, E3 e E4 (22,2%), “Agravos à saúde da criança quilombola”, quatro, E5, E6, E7 e E18 (22,2%), e “Cuidado com a criança quilombola”, um, E17 (5,6%).

No que se refere aos objetivos dos estudos, no eixo “Acesso aos serviços de saúde”, o E1 e o E2 buscam descrever como ocorre a assistência em nível de Atenção Primária à Saúde, enquanto que E3 e E4 pretendem entender o caminho percorrido até essa assistência e os fatores relacionados à frequência de comparecimento. Todos os estudos do eixo indicam relação direta com pelo menos cinco ODS, a saber: ODS 1: Erradicação da pobreza; ODS 3: Saúde e bem-estar; ODS 4: Educação de qualidade; ODS 6: Água potável e saneamento; e ODS 10: Redução das desigualdades^(3)^.

No eixo “Aspectos nutricionais da criança quilombola”, os estudos propõem avaliar o consumo alimentar (E9, E11 e E17), a segurança alimentar (E13 e E14), as condições nutricionais e socioeconômicas (E10 e E15) e a relação entre a alimentação e a ocorrência de doenças (E16). Nessa categoria, fica evidente a estreita relação entre a maioria dos estudos, principalmente com o ODS 1: Erradicação da pobreza, ODS 3: Saúde e bem-estar e ODS 6: Água limpa e saneamento.

Em relação aos resultados no eixo “Agravos à saúde da criança quilombola”, os estudos apresentaram prevalência de anemia em crianças menores de 2 anos associada ao maior número de moradores no domicílio (E6), e o inquérito realizado entre 2008 e 2018 (E7) mostrou que, apesar da redução dos casos, ainda se configura grave problema de saúde nessa faixa populacional.

No E5, constatou-se que, quanto maiores as desigualdades socioeconômicas, maiores são as taxas de mortalidade nas comunidades quilombolas, e que nessas comunidades essas taxas estão acima da média nacional. Nesse eixo, percebe-se nitidamente a adequação aos ODS 1: Erradicação da pobreza, ODS 3: Saúde e bem-estar e ODS 10: Redução das desigualdades.

O E18, pertencente ao eixo “Cuidado à criança quilombola”, detalha, por meio de um estudo do tipo exploratório-descritivo, a percepção dos cuidadores em relação à assistência oferecida pela Atenção Primária à Saúde, e tem como principais resultados o caráter prioritário que é dado por esses cuidadores à prática da medicina tradicional e sua combinação como o modelo biomédico oferecido pela Atenção Primária à Saúde. Nesse eixo temático, tem-se evidenciada a relação com o ODS 3 e o ODS 10.

## DISCUSSÃO

De acordo com os estudos analisados para esta revisão, a criança quilombola pode ser associada diretamente com alguns dos ODS. O ODS 1 visa erradicar a pobreza em todas as suas formas, em todos os lugares, pois muitas crianças quilombolas vivem em situações de pobreza com acesso limitado a recursos básicos. O ODS 3 trata da saúde e bem-estar de todas as idades, considerando possíveis disparidades como falta de acesso à atenção básica e precárias condições de saneamento básico. Evidencia-se que esse objetivo revela os desafios que marcam especialmente as crianças quilombolas no Brasil em relação ao acesso à saúde, a barreira geográfica (distância das instituições de saúde). Além da infraestrutura, recursos, discriminação e, especialmente, os aspectos nutricionais, observa-se que IA é um problema grave e prevalente. O ODS 4 foca em educação de qualidade para todos, considerando potenciais desafios por desigualdades estruturais e culturais. O ODS 6 refere-se à água potável e saneamento para todos, devido à falta de recursos das famílias e falta de investimento governamental. O ODS 10 trata da redução das desigualdades, pois as comunidades quilombolas, incluindo as crianças, frequentemente enfrentam dificuldades^(32,33)^.

A Estratégia para o Acesso Universal à Saúde e a Cobertura Universal de Saúde reconhece a importância de serviços de saúde de qualidade que atendam às necessidades de saúde das pessoas e comunidades. Isso envolve atenção especial à diversidade e às pessoas e populações em condições de vulnerabilidade. Os ODS 1, 3 e 10, consoantes com os estudos apresentados nesta revisão, estão relacionados à promoção de serviços de saúde de qualidade. ODS 1 combate a pobreza; ODS 3 promove ações de saúde; e ODS 10 reduz as desigualdades econômicas, sociais e políticas^(34)^. Os agravos à saúde de crianças quilombolas relacionados a fatores econômicos, sociais e culturais, como doenças, desnutrição e altas taxas de mortalidade, têm potencial para causar atraso no crescimento e desenvolvimento dessas crianças.

Em estudo que trata das situações de urgência e emergência pediátricas, tem-se como demanda que os serviços de saúde tenham boas condições e resposta rápida, além da garantia de acesso a ele pela população. Na comunidade quilombola, esse acesso se mostra dificultado por uma série de fatores, como distanciamento geográfico, condições socioeconômicas desfavoráveis e barreiras culturais. Assim, apresenta direta correlação com o ODS 3, mais especificamente a meta 3.8, pois destaca a importância de garantir o acesso integral e facilitado aos serviços de saúde^(3)^.

O ODS 3 tem como uma das metas até 2030: “Reduzir em um terço a mortalidade prematura por doenças não transmissíveis (DNT) por meio de prevenção e tratamento e promover a saúde mental e o bem-estar”^(33)^. Embora a anemia não seja uma doença não transmissível, a redução da prevalência de anemia em crianças quilombolas de Alagoas, Brasil, pode ser considerada um passo importante para alcançar a meta do ODS 3, pois a anemia persiste como um problema de saúde pública, especialmente entre as crianças de 6 a 24 meses.

O acesso às comunidades é realizado por estradas vicinais, sendo alguns trechos de difícil percurso, sobretudo no período chuvoso, quando se intensificam os buracos e a lama. Nesse contexto, pode-se afirmar que a qualidade das estradas está diretamente ligada às questões de saúde pública, educação e lazer, de modo que sua manutenção deve estar sempre nas pautas de desenvolvimento dos gestores municipais^(35)^.

A enfermagem tem um papel importante para o ODS 3, que busca garantir uma vida saudável e promover o bem-estar para todos em todas as idades. Na equipe multidisciplinar, a enfermagem é essencial para a promoção da saúde e a prevenção de doenças, contribuindo para o alcance de metas relacionadas à mortalidade infantil, saúde materna e controle de doenças. Isso também envolve a redução da desnutrição infantil, que afeta diretamente o bem-estar, a longevidade e a qualidade de vida da população mundial, principalmente das crianças quilombolas, que sofrem com a falta de acesso a recursos básicos, como água potável, saneamento e alimentação^(36)^.

Ademais, os profissionais de saúde, em especial os enfermeiros, como parte integrante do complexo sistema socioambiental mundial, são chamados a contribuir com todos os esforços possíveis para o alcance das ODS^(8)^. A enfermagem pode atuar na valorização dos saberes e práticas tradicionais das comunidades quilombolas, fortalecendo a atenção básica. No tocante às crianças quilombolas, tem intrínseca relação com as ODS 3, promovendo a saúde e bem-estar, ODS 4, promovendo educação de qualidade, nesse caso, educação em saúde, ODS 6, garantindo a disponibilidade e a gestão sustentável da água e saneamento para todos, e ODS 10, reduzindo as desigualdades referentes à falta de acesso aos serviços de saúde, por exemplo^(37)^.

Em outro estudo que trata da desnutrição infantil, também se verifica correlação com essa meta, além de apontar outros agravantes, que são a prevalência da desnutrição crônica e de obesidade. A Vigilância da Segurança Alimentar e Nutricional (II VIGISAN) descreve as condições de segurança alimentar e os níveis de IA dos domicílios das Unidades Federativas do Brasil. As análises abrangem uma seleção de 12.745 domicílios, localizados em áreas urbanas e rurais. Há proporção de IA moderada e grave, acima de 30,0%, nos domicílios com presença de menores de 10 anos^(38)^.

A mortalidade infantil evitável e as desigualdades sociais são grandes desafios enfrentados pelos países de baixa e média e renda (PBMRs). As diferenças na morbimortalidade vêm sendo amplamente discutidas entre grupos, como etnia, sexo, local de residência e nível socioeconômico^(39)^. Embora a relação com a desnutrição infantil não seja direta, a desnutrição é um dos principais fatores que contribuem para a mortalidade infantil em menores de 5 anos. Portanto, a redução da desnutrição é um passo importante para o alcance do ODS 3^(40)^.

A relação com o ODS 3, presente nos estudos que tratam de IA nas comunidades quilombolas, é que a fome e a IA estão associadas ao baixo desempenho escolar e comportamental em crianças, e são responsáveis por mais de 50% de todas as mortes em menores de 5 anos. Para alcançar os ODS de abolir a fome até 2030, é necessária a implantação de métodos adequados de saúde pública e políticas que garantam o acesso a alimentos nutritivos^(40)^.

Assegurar a disponibilidade e gestão sustentável da água e saneamento para todas e todos é a meta central do ODS 6, e está presente em estudos que tratam da IA nas crianças quilombolas, visto que, em muitas dessas comunidades, apenas um quarto dos domicílios possuía coleta de lixo, e um número reduzido, abastecimento de água e esgotamento sanitário. Dessa maneira, opõem-se ao indicador 6.1: até 2030, alcançar o acesso universal e equitativo à água potável e segura para todos^(41)^.

Relação semelhante ocorre em estudo que verificou a ocorrência de enteroparasitoses em crianças quilombolas, demonstrando sua íntima relação com as condições socioambientais, contaminação da água e dos alimentos consumidos. As condições socioeconômicas e de saneamento básico são consistentes em outras pesquisas. Os processos históricos de segregação e discriminação fizeram com que essas comunidades herdassem desvantagens. Essas descobertas estão alinhadas com a necessidade de atingir a meta 6.2 dos ODS, alcançar o acesso a saneamento e higiene adequados e equitativos para todos, e acabar com a defecação a céu aberto, especialmente para aqueles em situações vulneráveis^(7,41)^.

Desde o período da escravatura até os dias atuais, as estimativas das condições materiais de vida e saúde dos negros em relação aos brancos são piores, caracterizadas por pior infraestrutura sanitária e de saúde nos lugares em que vivem, falta de acesso a equipamentos e às instituições sociais, tais como escola, renda e serviços de saúde. Com o objetivo de reduzir a desigualdade dentro dos países e entre eles, a meta 10, em um de seus indicadores, pretende até 2030 empoderar e promover a inclusão social, econômica e política de todos, independentemente da idade, gênero, deficiência, raça, etnia, origem, religião, condição econômica ou outra^(42)^.

Contribuindo para a consecução dos ODS, a Atenção Primária à Saúde é considerada a melhor maneira de garantir melhorias sustentáveis nos resultados de saúde, sociedade e ambiente, quando apoiada por fortes políticas públicas alinhadas com as esferas econômica, política e social. O relatório das ODS destaca que, embora tenha havido progresso em algumas áreas, ainda existem grandes desafios a serem superados. Esses desafios e compromissos estão inter-relacionados e exigem soluções integradas. É imperativo adotar uma visão holística da Agenda 2030 e identificar as áreas mais afetadas para realizar intervenções direcionadas. Nesse sentido, podem surgir oportunidades valiosas para acelerar o progresso por meio da análise das inter-relações entre os objetivos^(43,44)^.

Diante desse cenário de injustiça, os quilombolas resistem e buscam manter suas redes de solidariedade, suas tradições e a relação com a natureza. Dessa maneira, essas características históricas, sociais e culturais estão relacionadas aos processos de adoecimento e saúde da população quilombola^(45,46)^. Ademais, a crescente população humana tem demandado o desenvolvimento acelerado de infraestrutura, como habitação, saúde, estradas de qualidade, educação, comunicação e serviços correlatos. Os estilos de vida tradicionais vistos nas comunidades quilombolas estão sendo gradativamente substituídos por padrões de vida impulsionados pela tecnologia^(47)^.

### Limitações do estudo

O número reduzido de publicações a nível internacional relacionadas à criança quilombola pode ter limitado as discussões apresentadas na revisão. Além disso, mesmo na literatura nacional, a maioria dos estudos encontrados não se refere especificamente a essa faixa etária.

### Contribuições para as áreas da saúde, enfermagem ou políticas públicas

A relevância do estudo para a saúde, sobretudo, para a enfermagem, reside no fato de apresentar um possível indicativo da grande possibilidade de se desenvolver mais pesquisas com a comunidade quilombola. A enfermagem, principalmente com sua ampla experiência em etnografia, tem todas as ferramentas necessárias para elaboração de estudos que irão contribuir com o avanço das políticas públicas voltadas para essa população, incluindo as crianças.

## CONCLUSÕES

Conclui-se que este estudo proporcionou o mapeamento de estudos de suma importância sobre a saúde de crianças quilombolas e a relação com os ODS. Com isso, clarificou a compreensão de quais temas relacionados às crianças quilombolas estão sendo abordados e debatidos. Por meio desse levantamento nas bases de dados nacionais e internacionais e uso de várias combinações de descritores, pôde-se inferir que a literatura produzida gira em torno de temas mais relacionados aos eixos definidos neste artigo e que possuem relação direta com os ODS.
